# New-Onset Status Epilepticus and Severe Behavioural Dysfunction in Anti-Gamma-Aminobutyric Acid B Receptor Encephalitis

**DOI:** 10.7759/cureus.105955

**Published:** 2026-03-27

**Authors:** Min Huang Chua, Yuen Kang Chia

**Affiliations:** 1 Internal Medicine, Hospital Queen Elizabeth, Kota Kinabalu, MYS; 2 Neurology, Hospital Queen Elizabeth, Kota Kinabalu, MYS

**Keywords:** autoimmune encephalitis, behavioural, cyclophosphamide, gaba(b)r, status epilepticus

## Abstract

The autoimmune encephalitides are an increasingly recognised group of neurological disorders. Among them, anti-gamma-aminobutyric acid B receptor (anti-GABA(B)R) encephalitis is a severe, frequently paraneoplastic autoimmune syndrome characterised by early, refractory seizures and prominent limbic dysfunction. Because initial neuroimaging and cerebrospinal fluid studies may be unremarkable, early cognitive and psychiatric manifestations are frequently misattributed to primary psychiatric disorders or adverse effects of anti-seizure medications. Here, we report the first case of a patient with anti-GABA(B)R encephalitis who first presented with the common symptom of a seizure in the state of Sabah, Malaysia.

## Introduction

The autoimmune encephalitides are an important and increasingly recognised group of neurological disorders affecting mainly the limbic system. Among them, anti-gamma-aminobutyric acid B receptor (anti-GABA(B)R) encephalitis is a severe, frequently paraneoplastic autoimmune syndrome characterised by early, refractory seizures and prominent limbic dysfunction [1].

Anti-GABA(B)R are found throughout the brain and spinal cord. However, the highest levels of anti-GABA(B)R are found mainly in the hippocampus, thalamus, and cerebellum [2]. Many patients with anti-GABA(B)R encephalitis thus develop seizures, behavioural dysfunction, and sometimes ataxia. This disease was first described by Lancaster et al. in 2010 [1], where almost half of the patients had an underlying malignancy, of which more than half were small cell lung carcinomas. It is postulated that these tumours may express anti-GABA(B)R, which may trigger an immune response against these receptors, causing symptoms.

In Malaysia, autoimmune encephalitides are increasingly being reported. More than 20 cases of anti-N-methyl-D-aspartate receptor (anti-NMDAR) encephalitis have been reported thus far [3,4]. However, anti-GABA(B)R encephalitis is still infrequently reported. Abdullah et al. in 2020 reported a case of anti-GABA(B)R encephalitis in a patient with Parkinson's disease [5]. To our best knowledge, thus far, no cases have been reported in Sabah, Malaysia.

The state of Sabah is located in East Malaysia, where the population consists predominantly of Austronesians. In 2021, Wong et al. reported that autoimmune encephalitides (especially anti-NMDAR antibody encephalitis) may be more common in Austronesians than in predominantly Caucasian populations in Europe [6].

We hereby present the first report of a case of anti-GABA(B)R encephalitis in an indigenous patient located in Kota Kinabalu, Sabah, Malaysia.

## Case presentation

A 46-year-old previously healthy Dusun (indigenous ethnic group in Sabah, Malaysia) man presented with a first-episode unprovoked generalised tonic-clonic (GTC) seizure. Initial neurological examination, basic metabolic panel, and a non-contrast computed tomography (CT) of the brain were normal. His cognition remained intact, and he was discharged without anti-seizure medication (ASM) after two days of observation in the ward.

Five days post-discharge, he presented again to the emergency department with recurrent GTC seizures. An electroencephalogram (EEG), however, did not reveal any abnormalities. He was then discharged on levetiracetam.

One week later, he re-presented yet again with seizures, progressive anterograde amnesia, and an inability to perform occupational tasks. It was thought at this point that this might have been related to levetiracetam, which prompted a switch to sodium valproate.

**Figure 1:**
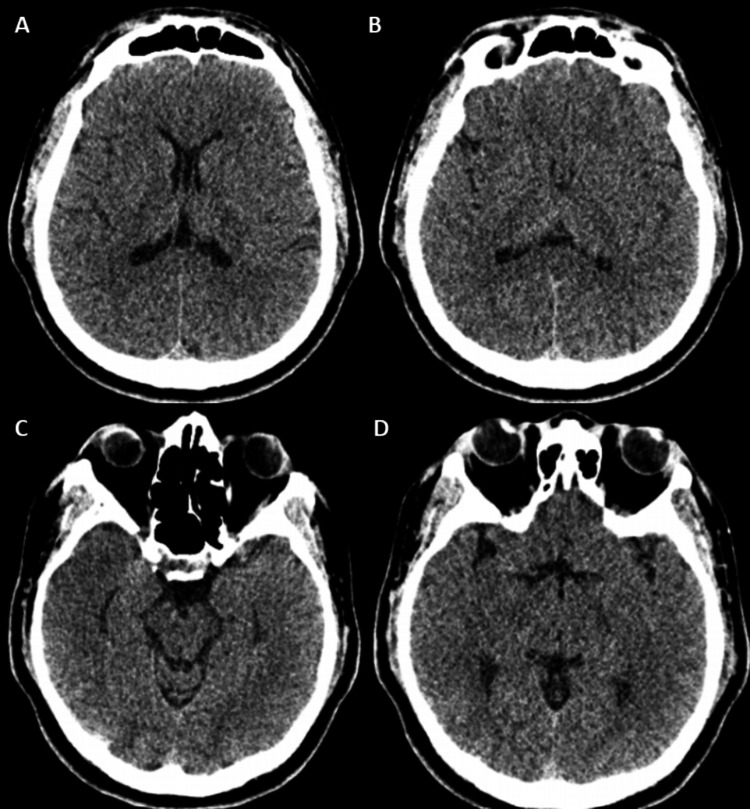
Plain CT of the brain did not reveal any abnormalities. (A-D) No significant abnormalities were detected. CT: computed tomography

**Figure 2:**
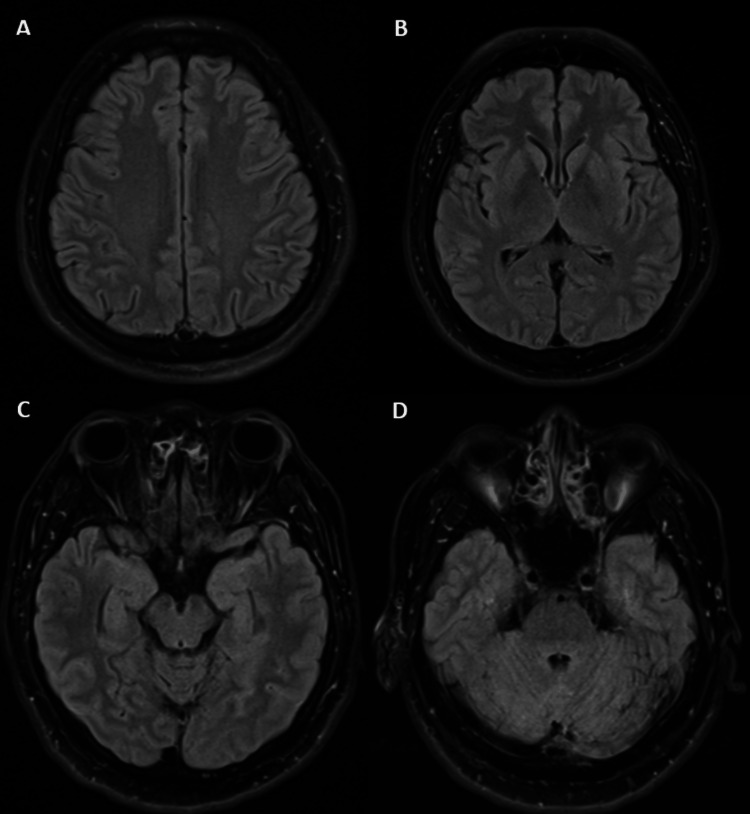
MRI T2-FLAIR of the patient did not show any significant limbic system changes. (A-D) No significant abnormalities were detected. MRI T2-FLAIR: magnetic resonance imaging T2-weighted fluid-attenuated inversion recovery

While re-evaluation of basic blood work, plain CT of the brain (Figure 1), and magnetic resonance imaging (MRI) of the brain revealed no significant abnormalities (Figure 2), the cerebrospinal fluid (CSF) analysis revealed a neuroinflammatory profile with elevated protein (0.78 g/L; reference range: 0.15-0.40 g/L) and a CSF/serum glucose ratio of 0.61 (reference range: 0.5-0.8) with a normal cell count. CSF infective workup including culture, cryptococcal antigen, tuberculosis GeneXpert, and polymerase chain reaction (PCR) panels for varicella-zoster, herpes simplex (1, 2, 6), *Neisseria meningitidis*, and *Listeria* were negative. The CSF sample was also outsourced to a private laboratory simultaneously for an autoimmune encephalitides antibody panel screen.

On day 4 of admission, he developed status epilepticus requiring endotracheal intubation and mechanical ventilation. A repeat 20-minute EEG during this episode showed mild to moderate cortical dysfunction with generalised theta slowing at 6-7 Hertz intermixed with intermittent alpha activities. No clinical seizure and epileptic discharge were seen during the EEG. Empirical antimicrobial therapy for meningoencephalitis was initiated alongside escalating ASMs (lamotrigine and sodium valproate), which resulted in adequate seizure control. 

Three days later, following extubation, the patient showed severe behavioural dysfunction, which included disruptive behaviour, agitation, and irritability. These neuropsychiatric symptoms proved refractory to standard pharmacological interventions including haloperidol and olanzapine. Levetiracetam was also restarted after the previous misattribution of behavioural symptoms had been excluded. However, his GTC seizures persisted despite conventional ASM therapies, albeit less frequently.

Subsequently, the outsourced CSF autoimmune panel returned positive for anti-GABA(B)R antibodies via indirect immunofluorescence assay (IFA) after around one week. He was immediately commenced on 1 g of intravenous methylprednisolone for five days, from which he derived no clinical benefit. He then received five cycles of therapeutic plasma exchange (PLEX), which resulted in a notable improvement in his seizure control. However, he still exhibited residual behavioural symptoms, such as an inability to concentrate and behavioural changes compared to his baseline.

He was subsequently given intravenous cyclophosphamide (600 mg/m²), which led to a complete resolution of his neuropsychiatric symptoms and seizures. Rituximab, while preferred, was not given due to institutional financial constraints.

Systemic malignancy screening, including a CT of the thorax, abdomen, and pelvis, showed no evidence of malignancy. He is scheduled to undergo a fluorodeoxyglucose-positron emission tomography (FDG-PET) scan at another institution. The patient was discharged on oral prednisolone and levetiracetam, with a plan for monthly cyclophosphamide infusions for six cycles.

At his one-month follow-up, he maintained normal cognitive function and seizure freedom and had successfully resumed full-time employment.

## Discussion

Anti-GABA(B)R encephalitis is a rare but highly morbid autoimmune condition characterised by seizures which can be refractory, severe cognitive impairment including anterograde amnesia, and psychiatric disturbances [1].

This is the first report of anti-GABA(B)R encephalitis in an indigenous adult in Sabah, Malaysia. This case underscores the diagnostic and subsequent therapeutic paradigms in the case of adult-onset unprovoked seizures.

The diagnosis of anti-GABA(B)R encephalitis can prove to be challenging, as initial neuroimaging can be radiologically occult, as seen in this patient [7]. A normal MRI of the brain is seen in approximately 40% of patients with anti-GABA(B)R encephalitis at presentation [8]. However, in adult patients presenting with new-onset seizures, the coexistence of profound anterograde amnesia should prompt the suspicion of limbic system involvement. In the case of this patient, status epilepticus, limbic system symptoms, and a normal MRI scan prompted a CSF autoantibody screen to look for autoimmune encephalitis. This is further supported by the initial CSF study, which showed an aseptic neuroinflammatory picture. In fact, it has been reported that anti-GABA(B)R encephalitis commonly presents with an epilepsy phase before an encephalitic phase [9]. Our patient fulfilled the criteria for possible autoimmune encephalitis, namely, subacute onset, seizure, and exclusion of alternative cause initially, and later on the definite autoimmune encephalitis, specific disease criteria in accordance with Graus et al. [7].

The initial misattribution of the patient's progressive anterograde amnesia is a common pitfall, as levetiracetam is well documented to cause neuropsychiatric symptoms [10]. Although the initial symptoms of amnesia were reasonably blamed on levetiracetam, the presence of concurrent refractory seizures and severe behavioural dysfunction should prompt the suspicion of an underlying severe neurological disorder. This suspicion is supported later by the presence of a neuroinflammatory profile in the CSF and the persistence of seizure and behavioural symptoms despite the cessation of levetiracetam.

The most common CSF profile of patients with anti-GABA(B)R encephalitis is pleocytosis, normal to slightly elevated CSF protein, and a normal glucose [11]. Oligoclonal bands are found in more than half the cases [12]. However, these are not always seen, as in our patient; his CSF protein was raised, but there was no pleocytosis. 

The delayed diagnosis was further compounded by the lack of on-site CSF autoantibody testing. In our institution, these assays are outsourced to a laboratory in Kuala Lumpur, resulting in a turnaround time of up to six weeks. While private laboratories offer expedited processing and results, the cost of these tests is prohibitive for many of our patients, rendering this faster alternative frequently inaccessible.

In regard to management, anti-GABA(B)R encephalitis frequently proves refractory to standard ASMs, as the pathophysiology is an antibody-mediated blockade of the inhibitory receptors, leading to ineffective slow synaptic inhibition [1]. Conventional ASMs commonly target voltage-gated sodium and calcium channels or work on synaptic vesicle release. Consequently, they are largely ineffective in controlling seizures without concurrent immunomodulation to stop the underlying antibody-mediated process. In this patient, despite conventional ASMs, he still progressed to status epilepticus.

For the intervention to be effective, it must aggressively address the underlying autoimmune process. While first-line therapies including intravenous methylprednisolone and therapeutic PLEX are highly effective at reducing circulating autoantibodies and providing acute clinical stabilisation, they do not stop the production of autoantibodies. In our case, persistent behavioural dysfunction after intravenous methylprednisolone and PLEX necessitated second-line therapy. The administration of intravenous cyclophosphamide proved to be effective in achieving complete neurological recovery [13]. Although clinical escalation protocols are predominantly derived from anti-NMDAR antibody encephalitis, where approximately 50% of patients fail first-line therapy, this paradigm is standardly applied to other autoimmune encephalitides [14]. 

The search for the cause of anti-GABA(B)R encephalitis is inextricably linked to paraneoplastic aetiology. Approximately 50% of cases are associated with malignancy, especially small cell lung cancer (SCLC) [8]. SCLC cells can express anti-GABA(B)R ectopically, triggering a cross-reactive immune response. While this patient's initial CT of the thorax was negative, CT lacks the sensitivity to detect micro-lesions of early SCLC. The delayed acquisition of an FDG-PET scan represents a significant institutional limitation in our case.

Close monitoring, including repeating imaging every three to six months subsequently, followed by six-monthly for four years, remains essential to definitively rule out a paraneoplastic aetiology [15].

## Conclusions

Anti-GABA(B)R encephalitis must be suspected in adults presenting with new-onset unprovoked seizures progressing to status epilepticus, particularly when accompanied by profound limbic dysfunction. They are characterised by their rapid progression. Because clinical progression is catastrophic, an initial comprehensive CSF test is paramount to identify them so that treatment can be rapidly administered.

When initial first-line treatment fails, quick escalation of therapy can result in favourable outcomes. However, it must be noted that early treatment is essential to mitigate the risk of permanent neurological sequelae. A thorough hunt for an underlying neoplasm must be pursued as anti-GABA(B)R encephalitis is often associated with malignancy. Early detection and aggressive oncological management of the underlying malignancy remain the utmost prerequisites for neurological recovery and long-term survival.

## References

[1] Lancaster E, Lai M, Peng X (2010). Antibodies to the GABAB receptor in limbic encephalitis with seizures: case series and characterisation of the antigen. Lancet Neurol.

[2] Fritschy JM, Meskenaite V, Weinmann O, Honer M, Benke D, Mohler H (1999). GABAB-receptor splice variants GB1a and GB1b in rat brain: developmental regulation, cellular distribution and extrasynaptic localization. Eur J Neurosci.

[3] Abdullah S, Lim SY, Goh KJ, Lum L, Tan CT (2011). Anti-N-methyl-D-aspartate receptor (NMDAR) encephalitis: a series of ten cases from a university hospital in Malaysia. Neurol. Asia.

[4] Sim ST, Chia PK, Chhoa KH, Chai BS (2021). Anti-N-methyl-D-aspartate receptor (NMDAR) encephalitis in Malaysia: a review article. Malays J Med Health Sci.

[5] Abdullah NS, Jan TH, Remli R, Mukari SA, Ibrahim NM (2020). Anti-GABAB receptor encephalitis presenting with atypical corticobasal syndrome in a patient with Parkinson's disease. J Mov Disord.

[6] Wong CK, Hor JY, Loo YP (2021). High incidence of NMDAR encephalitis among Austronesians: a population-based study in Sabah, Malaysia. J Neuroimmunol.

[7] Graus F, Titulaer MJ, Balu R (2016). A clinical approach to diagnosis of autoimmune encephalitis. Lancet Neurol.

[8] Höftberger R, Titulaer MJ, Sabater L (2013). Encephalitis and GABAB receptor antibodies: novel findings in a new case series of 20 patients. Neurology.

[9] Maureille A, Fenouil T, Joubert B (2019). Isolated seizures are a common early feature of paraneoplastic anti-GABAB receptor encephalitis. J Neurol.

[10] Verrotti A, Prezioso G, Di Sabatino F, Franco V, Chiarelli F, Zaccara G (2015). The adverse event profile of levetiracetam: a meta-analysis on children and adults. Seizure.

[11] Zhu F, Shan W, Lv R, Li Z, Wang Q (2020). Clinical characteristics of anti-GABA-B receptor encephalitis. Front Neurol.

[12] Guan HZ, Ren HT, Yang XZ (2015). Limbic encephalitis associated with anti-γ-aminobutyric acid B receptor antibodies: a case series from China. Chin Med J (Engl).

[13] Abboud H, Probasco JC, Irani S (2021). Autoimmune encephalitis: proposed best practice recommendations for diagnosis and acute management. J Neurol Neurosurg Psychiatry.

[14] Titulaer MJ, McCracken L, Gabilondo I (2013). Treatment and prognostic factors for long-term outcome in patients with anti-NMDA receptor encephalitis: an observational cohort study. Lancet Neurol.

[15] Titulaer MJ, Soffietti R, Dalmau J (2011). Screening for tumours in paraneoplastic syndromes: report of an EFNS task force. Eur J Neurol.

